# Supplementary material for: OpenMM 7: Rapid development of high performance algorithms for molecular dynamics
Source: PLoS Comput Biol. 2017 Jul 26;13(7):e1005659. doi: 10.1371/journal.pcbi.1005659 (PMC5549999; doi:10.1371/journal.pcbi.1005659)
Supplement: S1 Source Code — (ZIP) [file pcbi.1005659.s002.zip › OpenMM-7.0.1-Source/docs/api-c++/generated/OpenMM.CustomAngleForce.html]

CustomAngleForce — OpenMM @OPENMM\_MAJOR\_VERSION@.@OPENMM\_MINOR\_VERSION@ documentation


# CustomAngleForce¶

*class* `OpenMM::``CustomAngleForce`¶
:   This class implements interactions between sets of three particles that depend on the angle between them. Unlike `HarmonicAngleForce`, the functional form of the interaction is completely customizable, and may involve arbitrary algebraic expressions. In addition to the angle formed by the particles, it may depend on arbitrary global and per-angle parameters.

    To use this class, create a `CustomAngleForce` object, passing an algebraic expression to the constructor that defines the interaction energy between each set of particles. The expression may depend on theta, the angle formed by the particles, as well as on any parameters you choose. Then call `addPerAngleParameter()` to define per-angle parameters, and `addGlobalParameter()` to define global parameters. The values of per-angle parameters are specified as part of the system definition, while values of global parameters may be modified during a simulation by calling `Context::setParameter()`. Finally, call `addAngle()` once for each angle. After an angle has been added, you can modify its parameters by calling `setAngleParameters()`. This will have no effect on Contexts that already exist unless you call `updateParametersInContext()`.

    As an example, the following code creates a `CustomAngleForce` that implements a harmonic potential:

    ```
    CustomAngleForce* force = new CustomAngleForce("0.5*k*(theta-theta0)^2");
    ```

    This force depends on two parameters: the spring constant k and equilibrium angle theta0. The following code defines these parameters:

    ```
    force->addPerAngleParameter("k");
    force->addPerAngleParameter("theta0");
    ```

    Expressions may involve the operators + (add), - (subtract), \* (multiply), / (divide), and ^ (power), and the following functions: sqrt, exp, log, sin, cos, sec, csc, tan, cot, asin, acos, atan, sinh, cosh, tanh, erf, erfc, min, max, abs, floor, ceil, step, delta, select. All trigonometric functions are defined in radians, and log is the natural logarithm. step(x) = 0 if x is less than 0, 1 otherwise. delta(x) = 1 if x is 0, 0 otherwise. select(x,y,z) = z if x = 0, y otherwise.

    Methods

    |  |  |
    | --- | --- |
    | `CustomAngleForce` | Create a `CustomAngleForce`. |
    | `getNumAngles` | Get the number of angles for which force field parameters have been defined. |
    | `getNumPerAngleParameters` | Get the number of per-angle parameters that the interaction depends on. |
    | `getNumGlobalParameters` | Get the number of global parameters that the interaction depends on. |
    | `getEnergyFunction` | Get the algebraic expression that gives the interaction energy for each angle |
    | `setEnergyFunction` | Set the algebraic expression that gives the interaction energy for each angle |
    | `addPerAngleParameter` | Add a new per-angle parameter that the interaction may depend on. |
    | `getPerAngleParameterName` | Get the name of a per-angle parameter. |
    | `setPerAngleParameterName` | Set the name of a per-angle parameter. |
    | `addGlobalParameter` | Add a new global parameter that the interaction may depend on. |
    | `getGlobalParameterName` | Get the name of a global parameter. |
    | `setGlobalParameterName` | Set the name of a global parameter. |
    | `getGlobalParameterDefaultValue` | Get the default value of a global parameter. |
    | `setGlobalParameterDefaultValue` | Set the default value of a global parameter. |
    | `addAngle` | Add an angle term to the force field. |
    | `getAngleParameters` | Get the force field parameters for an angle term. |
    | `setAngleParameters` | Set the force field parameters for an angle term. |
    | `updateParametersInContext` | Update the per-angle parameters in a `Context` to match those stored in this `Force` object. |
    | `usesPeriodicBoundaryConditions` | Returns whether or not this force makes use of periodic boundary conditions. |

    `CustomAngleForce`(*const* std::string &*energy*)¶
    :   Create a `CustomAngleForce`.

        |  |  |
        | --- | --- |
        | Parameters: |  |

        - **energy** – an algebraic expression giving the interaction energy between three particles as a function of theta, the angle between them

    int `getNumAngles`() *const*¶
    :   Get the number of angles for which force field parameters have been defined.

    int `getNumPerAngleParameters`() *const*¶
    :   Get the number of per-angle parameters that the interaction depends on.

    int `getNumGlobalParameters`() *const*¶
    :   Get the number of global parameters that the interaction depends on.

    *const* std::string &`getEnergyFunction`() *const*¶
    :   Get the algebraic expression that gives the interaction energy for each angle

    void `setEnergyFunction`(*const* std::string &*energy*)¶
    :   Set the algebraic expression that gives the interaction energy for each angle

    int `addPerAngleParameter`(*const* std::string &*name*)¶
    :   Add a new per-angle parameter that the interaction may depend on.

        |  |  |
        | --- | --- |
        | Parameters: |  |

        - **name** – the name of the parameter

        |  |  |
        | --- | --- |
        | Returns: | the index of the parameter that was added |

    *const* std::string &`getPerAngleParameterName`(int *index*) *const*¶
    :   Get the name of a per-angle parameter.

        |  |  |
        | --- | --- |
        | Parameters: |  |

        - **index** – the index of the parameter for which to get the name

        |  |  |
        | --- | --- |
        | Returns: | the parameter name |

    void `setPerAngleParameterName`(int *index*, *const* std::string &*name*)¶
    :   Set the name of a per-angle parameter.

        |  |  |
        | --- | --- |
        | Parameters: |  |

        - **index** – the index of the parameter for which to set the name
        - **name** – the name of the parameter

    int `addGlobalParameter`(*const* std::string &*name*, double *defaultValue*)¶
    :   Add a new global parameter that the interaction may depend on.

        |  |  |
        | --- | --- |
        | Parameters: |  |

        - **name** – the name of the parameter
        - **defaultValue** – the default value of the parameter

        |  |  |
        | --- | --- |
        | Returns: | the index of the parameter that was added |

    *const* std::string &`getGlobalParameterName`(int *index*) *const*¶
    :   Get the name of a global parameter.

        |  |  |
        | --- | --- |
        | Parameters: |  |

        - **index** – the index of the parameter for which to get the name

        |  |  |
        | --- | --- |
        | Returns: | the parameter name |

    void `setGlobalParameterName`(int *index*, *const* std::string &*name*)¶
    :   Set the name of a global parameter.

        |  |  |
        | --- | --- |
        | Parameters: |  |

        - **index** – the index of the parameter for which to set the name
        - **name** – the name of the parameter

    double `getGlobalParameterDefaultValue`(int *index*) *const*¶
    :   Get the default value of a global parameter.

        |  |  |
        | --- | --- |
        | Parameters: |  |

        - **index** – the index of the parameter for which to get the default value

        |  |  |
        | --- | --- |
        | Returns: | the parameter default value |

    void `setGlobalParameterDefaultValue`(int *index*, double *defaultValue*)¶
    :   Set the default value of a global parameter.

        |  |  |
        | --- | --- |
        | Parameters: |  |

        - **index** – the index of the parameter for which to set the default value
        - **defaultValue** – the default value of the parameter

    int `addAngle`(int *particle1*, int *particle2*, int *particle3*, *const* std::vector<double> &*parameters* = std::vector< double >())¶
    :   Add an angle term to the force field.

        |  |  |
        | --- | --- |
        | Parameters: |  |

        - **particle1** – the index of the first particle connected by the angle
        - **particle2** – the index of the second particle connected by the angle
        - **particle3** – the index of the third particle connected by the angle
        - **parameters** – the list of parameters for the new angle

        |  |  |
        | --- | --- |
        | Returns: | the index of the angle that was added |

    void `getAngleParameters`(int *index*, int &*particle1*, int &*particle2*, int &*particle3*, std::vector<double> &*parameters*) *const*¶
    :   Get the force field parameters for an angle term.

        |  |  |
        | --- | --- |
        | Parameters: |  |

        - **index** – the index of the angle for which to get parameters
        - **particle1** – [out] the index of the first particle connected by the angle
        - **particle2** – [out] the index of the second particle connected by the angle
        - **particle3** – [out] the index of the third particle connected by the angle
        - **parameters** – [out] the list of parameters for the angle

    void `setAngleParameters`(int *index*, int *particle1*, int *particle2*, int *particle3*, *const* std::vector<double> &*parameters* = std::vector< double >())¶
    :   Set the force field parameters for an angle term.

        |  |  |
        | --- | --- |
        | Parameters: |  |

        - **index** – the index of the angle for which to set parameters
        - **particle1** – the index of the first particle connected by the angle
        - **particle2** – the index of the second particle connected by the angle
        - **particle3** – the index of the third particle connected by the angle
        - **parameters** – the list of parameters for the angle

    void `updateParametersInContext`(Context &*context*)¶
    :   Update the per-angle parameters in a `Context` to match those stored in this `Force` object. This method provides an efficient method to update certain parameters in an existing `Context` without needing to reinitialize it. Simply call `setAngleParameters()` to modify this object’s parameters, then call `updateParametersInContext()` to copy them over to the `Context`.

        This method has several limitations. The only information it updates is the values of per-angle parameters. All other aspects of the `Force` (such as the energy function) are unaffected and can only be changed by reinitializing the `Context`. The set of particles involved in a angle cannot be changed, nor can new angles be added.

    bool `usesPeriodicBoundaryConditions`() *const*¶
    :   Returns whether or not this force makes use of periodic boundary conditions.

        |  |  |
        | --- | --- |
        | Returns: | false |

High performance molecular simulation on GPUs


### Navigation

- Core Objects
- Forces
- Integrators
- Extras

©2015, Stanford University and the Authors.
|
Powered by Sphinx 1.3.1
& Alabaster 0.7.7
|
Page source
